# Rigg's Disease

**Published:** 1885-09

**Authors:** G. A. Mills


Page(s) missing hard and soft tissues, so that their attachments with the roots were gradually being destroyed. His knowledge of surgical principles suggested a practical application to these diseased localities, and he proceeded to the removal of all foreign substances from the roots of teeth, and the trimming of the necrosed edge of the alveolus to the life-line, leaving nature to restore to a normal condition. Dr. Rigg's views naturally excited a variety of comment—some expressing disbelief, and others accepting his novel ideas and statements. Not a few denied the existence of a necrosed edge of the alveolus. Dr. Riggs had devised a set of instruments well adapted for the treatment of this disorder—and these were unique and new, yet there was an effort on the part of a very few to dispute his claim to this invention; this did not prove a success. This body (the Connecticut Valley Dental Association) subsequently passed a resolution giving credit to Dr. Riggs for originality relative to the new pathology of the disorder now termed Riggs's disease, and so named at about that time in honor of Dr. Riggs. I have previously remarked that nothing of the doctor's views had ever been published so far as known. But—having become personally much interested in this disease, and in the discussion of it, and also finding my position regarding it misunderstood by several dentists—I was led to prepare a series of articles (six), which were published in the "Dental Cosmos" during the years 1876 and 1877, under the title of "What I know about Riggs's Disease," in one of which articles I challenged the record of views corresponding to Dr. Riggs's. Since then not a word has come from any source to show that he is antedated in the matter. I may add that a confirmation of his views and their acceptance by many members of the dental profession have gradually taken place. I am glad to say that to-day it is the most prominent subject for consideration before dentists generally. Only a limited number, however, have come to a correct understanding of what is required and how to meet the requirements. These few are demonstrating a successful treatment of the disorder. At this point of my article it seems advisable to introduce a feature which I shall elaborate later on; it is in reference to the technical term by which this disorder is now known—viz., *pericementitis*, substituted for the term well known by medical men—dental periostitis—meaning inflammation of the dental periosteum. This term (pericementitis) originated in the laboratory of Charles Heitzman, M. D., of New York city, during the late investigations made there by dentists under his instruction. The general subject of pericementitis it is not my design to discuss here, but it is necessary to make the distinction clear between Riggs's disease and general pericementitis. Riggs's disease is a peculiar phase of pericementitis; it may exist to the final loss of all the teeth, without a sign of any other phase of this disorder.

As the nature of this disease is so plainly embodied in my brief history of the matter which includes its pathology, it would seem that my readers need not be ignorant to its main features; therefore I pass to consider the diagnosis.

To diagnosticate an incipient case, or first manifestation, as it is often seen in the mouths of children (even at a very early age): The simplest form of the disease may often be seen at the peripheral part of the festoon of the gum tissue indicated by a congested appearance; by lifting this gum with a delicate instrument, there will be seen a little seed-like granule of calcific substance. Another case might show a deep red and raw-looking, elongated appearance of the gum-tissue about the necks of the teeth, and with or without any deposit; there may be also a looseness of the gum about the teeth, which causes quite a pocket. This latter condition is often a sequela of exanthematous disorders. The gums are often extremely sensitive to the touch. In the various cases we find general congestion, easy hemorrhage, pale and bloodless gums, a decidedly anæmic and frequently pimpled surface of the gums—the latter appearance in adults. Not uncommonly a first warning to the patient (adult) will be pain or tenderness about the tooth or teeth, and an examination will not reveal any decay, death of pulp (commonly called nerve), or evidence of inflammation of pulp. This is what I shall term a subtle manifestation, for it has been believed there could be no inflammation of the dental membrane without a disturbance of the pulp. This is now proved to be untrue, for abscesses do occur while the pulp remains normal. In a large proportion of cases there will be, on light pressure, a flow of pus from under the gums, and oftentimes it is a copious discharge. This may be general, or it may be confined to a single tooth. Looseness of one or more of the teeth may be observed; also malposition, and this commonly after an occluding tooth is lost. I have given in detail enough of the manifestations to lead one even superficially familiar with unhealthty conditions to the diagnosis. It will be observed that I have omitted other conditions of disease that are manifested in the mouth, associated with the teeth and allied structures—viz.: syphilis, salivation, and scurvy. While in some instances these may be separated from the disorder in question, yet they are sometimes complications. I will mention another marked diagnostic feature associated always with an active stage of the disorder, and that is the odor which is distinctly noticeable to one familiar with Riggs's disease. There are other local manifestations that are, without doubt, largely influenced by the disease, but are commonly classed as expressions of constitutional debility, and still they may be wholly the result of the disorder under definition. This is proved by the arresting of the disease when the disabilities referred to are removed. Recession of gum-tissue is often seen, and no apparent inflammatory condition. While this is a peculiar phase, I maintain it is the same disorder. My term for it is atrophy of the gum-tissue—erosion of the tooth-structure, causing grooves across and around the necks of the teeth, not infrequently taking a serpentine direction. This also is a manifestation of the same disorder, as it is arrested by the treatment which will now be described.

TREATMENT.—As the nature of the disorder has proved to be novel, so will the treatment appear, as Dr. Riggs was the inventor of a set of instruments with which to perform the operations required in treating the disease. Each one is six inches in length, including the handle, which is of ebony and steel, octagonal and tapered; the blades are seven-eighths of an inch long, bent at an obtuse angle. The instruments are in two pairs, and there are two single ones. One pair has a knife edge and a safe edge; the other pair has the same, but these are reversed in their bevels—made so for the purpose of working at a different angle of the mouth, and from the operator instead of toward him. The single ones are double knife-edged, and differing in thickness of blade. Perhaps no better idea can be given of the general form of the blades than to say they resemble the half of a snipe's bill, the long, ovoid point being particularly adapted to ferreting out the intricate and deep-seated disordered parts of the hard and soft tissues about the roots of the teeth. In their dimensions they may seem ponderous to a novice, but in the hands of an expert no instrument can be more efficiently and delicately used. It must now be seen, by the description and location of Dr. Riggs's disease, that most of the operation is under the gum-tissue and out of sight, so that necessarily to know when the operation is complete at a given point can only be accomplished by an acquired and acute sense of touch. It may be said that the Riggs's treatment has instituted a distinct and systematic mode of arresting the disease. Rightly understood and rightly practiced, I regard this treatment as the most efficient in dental surgery. The severity of the cases differs according to constitutional conditions, and, if the dentist is the doctor, he will know whether the patient can be wisely aided by constitutional treatment. The prognosis must be based upon the conditions as they appear in each case.

From an extensive experience within the last ten years in the treatment of a large number of cases, and the success attained, I am justified in saying that Riggs's disease can no longer be classed among the incurable ones.

It is perfectly plain that this disease is not confined to any one period in life. Under the age of forty I have had numerous cases in the most active stages of progress—so noticeable that there was almost spontaneous hemorrhage of the gums, and such an excessive flow of pus that the service of napkins for absorbing was required in sleeping hours. These facts can be testified to by well-known physicians. As one impressed with the prevalence of Riggs's disease, and its destructive effect on the general health, I should be remiss in duty if I were silent, or neglected to call the earnest attention of medical men and the public to the grave facts, for they have had too little consideration. I would say emphatically that the most serious complications may arise, and the worst septic conditions may be threatened and encountered, from pure neglect. That one disorder not arrested calls others of a more serious nature into existence is a well-known fact among medical men.—*New York Med. Journal.*

---

ARTICLE III.

### A GERMAN TRIAL AGAINST DENTISTS FOR USING THE TITLE "DOCTOR OF DENTAL SURGERY."

[Translated for American Journal of Dental Science.]

